# A Mixed Methods and Triangulation Model for Increasing the Accuracy of Adherence and Sexual Behaviour Data: The Microbicides Development Programme

**DOI:** 10.1371/journal.pone.0011600

**Published:** 2010-07-21

**Authors:** Robert Pool, Catherine M. Montgomery, Neetha S. Morar, Oliver Mweemba, Agnes Ssali, Mitzy Gafos, Shelley Lees, Jonathan Stadler, Angela Crook, Andrew Nunn, Richard Hayes, Sheena McCormack

**Affiliations:** 1 Centre for International Health Research (CRESIB), University of Barcelona, Barcelona, Spain; 2 HIV Prevention Research Unit, Medical Research Council, Durban, South Africa; 3 Department of Community Medicine, University of Zambia, Lusaka, Zambia; 4 MRC/UVRI Uganda Research Unit on AIDS, Medical Research Council UK/Uganda Virus Research Institute, Entebbe, Uganda; 5 Africa Centre for Health and Population Studies, University of KwaZulu-Natal, Mtubatuba, South Africa; 6 Infectious Disease Epidemiology Unit, London School of Hygiene and Tropical Medicine, London, United Kingdom; 7 Reproductive Health and HIV Research Unit, Witwatersrand University, Johannesburg, South Africa; 8 Medical Research Council Clinical Trials Unit, London, United Kingdom; 9 Centre for Global Health and Inequality, University of Amsterdam, Amsterdam, The Netherlands; Tulane University, United States of America

## Abstract

**Background:**

The collection of accurate data on adherence and sexual behaviour is crucial in microbicide (and other HIV-related) research. In the absence of a “gold standard” the collection of such data relies largely on participant self-reporting. After reviewing available methods, this paper describes a mixed method/triangulation model for generating more accurate data on adherence and sexual behaviour in a multi-centre vaginal microbicide clinical trial. In a companion paper some of the results from this model are presented [1].

**Methodology/Principal Findings:**

Data were collected from a random subsample of 725 women (7.7% of the trial population) using structured interviews, coital diaries, in-depth interviews, counting returned gel applicators, focus group discussions, and ethnography. The core of the model was a customised, semi-structured in-depth interview. There were two levels of triangulation: first, discrepancies between data from the questionnaires, diaries, in-depth interviews and applicator returns were identified, discussed with participants and, to a large extent, resolved; second, results from individual participants were related to more general data emerging from the focus group discussions and ethnography. A democratic and equitable collaboration between clinical trialists and qualitative social scientists facilitated the success of the model, as did the preparatory studies preceding the trial. The process revealed some of the underlying assumptions and routinised practices in “clinical trial culture” that are potentially detrimental to the collection of accurate data, as well as some of the shortcomings of large qualitative studies, and pointed to some potential solutions.

**Conclusions/Significance:**

The integration of qualitative social science and the use of mixed methods and triangulation in clinical trials are feasible, and can reveal (and resolve) inaccuracies in data on adherence and sensitive behaviours, as well as illuminating aspects of “trial culture” that may also affect data accuracy.

## Introduction

The accurate measurement of product use and related behaviour in microbicide trials (but also in many other fields) is important for a number of reasons.

First, poor adherence reduces the chance of demonstrating effectiveness. If a trial shows overall benefit then relating the level of protection to adherence is valuable in interpreting the results, and has important implications for predicting effectiveness in real-life settings. Also, in order to properly interpret the results of trials that do not show a protective effect, it is necessary to be able to identify to what extent this may be due to the product not being efficacious, participants not using it, or not using it correctly, participants increasing protective behaviours such as condom use, increased risky behaviour related to perceived protection of the product, or other high-risk behaviours such as anal sex [2], [3].

Second, the use of investigational microbicides may negatively affect participants, either directly as a result of harmful side effects or indirectly as a result of changes in behaviour. Having accurate data on product use and related behaviour is important for assessing safety [2], [3].

Third, understanding the reasons for different levels of adherence provides insights that are useful for the design of future clinical trials and for facilitating rollout and access if the product proves effective.

Finally, understanding the reasons for non-adherence and for not reporting or inaccurately reporting non-adherence and other relevant behaviours is also important because it can be fed back into the trial and used to improve adherence and the accuracy of adherence data. Similarly, understanding the issues involved in the inaccurate reporting of sexual behaviour and other relevant practices during the trial makes it possible to adjust data collection techniques and improve accuracy.

The assumption among biomedical researchers is that the best and most accurate measure of adherence (and other relevant behaviours) would be a validated biomarker – some objective biological indicator of whether the study product has been used or whether the participant has engaged in certain behaviours (such as condom use or unprotected sex). This could then be used as the “gold standard” against which the accuracy of other perhaps easier and cheaper methods could be measured. Unfortunately, although there are a number of potential biomarkers for both sexual behaviour and vaginal microbicide use, these have either not been adequately validated or are not feasible in large clinical trials due to issues such as cost, logistics and acceptability. So in the absence of a “gold standard”, the collection of data on adherence, sexual behaviour (including high-risk behaviours that are often stigmatised) and vaginal practices relevant to microbicide studies, relies largely on participant self-reporting, usually through structured questionnaires.

The limitations of structured questionnaires for collecting sexual behaviour and other sensitive data are well recognised. Also, because of the sensitivity of the topics and the likelihood of desirability bias, structured face-to-face interviews in a clinic setting are not ideal for collecting accurate data (if project staff promote condoms and ask participants to use gel every time they have sex, then participants are more likely to report that they have complied, and they are less likely to report stigmatised behaviours such as anal sex). They are also not ideal for understanding participants' reasons for non-adherence or the scope and reasons for inaccurate reporting. Various other methods, are available for collecting self-reported sexual behaviour data, but these methods also all have disadvantages. In recent years behavioural research relating to HIV has moved increasingly toward using and comparing different methods, and microbicide researchers have started to experiment with methods based on participant self-assessment techniques such as computer assisted self-interview (CASI). Usually these studies report on the fit between data from different self-report methods, or between self-report and biological data.

The use of mixed methods in a single study has often revealed inconsistencies between the data collected using different instruments, but not much attempt has been made to find out *why* results are inconsistent. The assumption seems to be that study participants are simply unreliable when it comes to reporting this kind of behaviour. There has also been no attempt to resolve the inconsistencies that emerge during the study: different methods are used, and the inconsistent results are only identified and discussed after the completion of the study.

What is required is to move beyond mere comparison to investigate and understand the reasons for divergent results, and then attempt to increase the accuracy of the results. This can be accomplished through the use of mixed methods and the triangulation of results, in dialogue with participants, and *during* the study. This paper reports on what is, as far as we are aware, the largest and most comprehensive use of mixed methods and triangulation in the context of medical research. It has generated a rich and unique set of data on adherence and sexual and other relevant behaviour.

In what follows we first review the pros and cons of the main methods currently available (or being developed) for measuring adherence and related behaviour in vaginal microbicide and similar studies. Then, after briefly describing the Microbicides Development Programme MDP301 Phase III trial, we describe in detail the mixed method/triangulation model that has been developed by the MDP team in an attempt to gather more accurate data on adherence and sexual behaviour. In a separate paper we discuss some of the findings from this process [1].

## Overview of Available Methods for Measuring Adherence and Sexual Behaviour

### Respondent-independent methods

Because all self-reporting is ultimately dependent on the truthfulness, memory and accuracy of the study participants, the development and use of respondent-independent methods is seen as a priority.

- **Biomarkers**. A biomarker has been defined as “a characteristic that is objectively measured and evaluated as an indicator of normal biological processes, pathogenic processes, pharmacological responses to a therapeutic intervention” [4]. In microbicide research the main focus is on biomarkers of semen exposure, cervicovaginal inflammation, HIV and STIs [5]. The interest is in both surrogate endpoints and the verification of behaviour. There are various candidate biomarkers for semen exposure [6] and if validated these could provide reliable evidence of unprotected intercourse. However, they would not provide information on other relevant behaviours (such as anal sex) or the reasons for behaviours, or generate much information on adherence (which would require a different biomarker). Pregnancy or HIV or STI infection are sometimes used as biomarkers of unprotected sex, but all they really tell us is that the participant had at least one unprotected sex act (and in the case of HIV infection there are other possible means of transmission).
- **Applicator stain test**. In the Carraguard vaginal gel efficacy trial, product adherence was assessed through self-reports, counting returned applicators and a staining test that was meant to show which applicators had been vaginally inserted [7], [8], [9]. However, the high proportion of empty applicators that were not confirmed by the stain test (39%) suggests that this technique may not have been adequately validated [10]. While potentially more reliable than self-reports, this method is time consuming, open to observer bias (the staining has to be interpreted), and is only proof that the applicator was inserted into a vagina, not that it has been used by the participant in question, or that the product was actually administered.
- **“Smart applicators”**. The International Partnership for Microbicides (IPM) is currently developing “smart applicators” that could register time, date and temperature when used, thus providing some verification of product use. However, such applicators would still miss essential information: they would be unable to tell us whose vagina they have been in, whether the participant had sex, what kind of partner she had, or the reasons for non-adherence.
- **Applicator returns**. Counting used product applicators is a relatively simple (though perhaps not really respondent-independent) method for assessing product use in microbicide trials, and investigators consider this a relatively reliable measure of adherence. However, it may be inconvenient for participants or they may be unwilling to return applicators, and it does not reveal whether the product was used as intended (for example the gel might have been squeezed out in order to return empty applicators, or it may have been used by someone else). Also, in order to estimate adherence to products that have to be inserted prior to sex, product counts still need to be related to self-reported sex acts and timing of use. This latter point also applies to the stain test and the smart applicator.

All respondent independent methods, but particularly biomarkers and smart applicators, also raise serious questions about trust and acceptability: how willing will study participants be to use products that have been designed on the assumption that they (the participants) are unreliable?

### Self-report methods

#### Face-to-face interviews

- **Structured interviews**, using pre-coded questionnaires or case record forms (CRF), have been the main instrument for collecting behavioural data in microbicide trials (and other medical research). They efficiently generate large standardised datasets that are relatively easy to manage and analyse. They are, however, inflexible and prone to desirability bias. Also, any systematic misunderstandings that occur during the interviews are hidden in the neat dataset that results.
- **Open or semi-structured in-depth interviews** are more flexible and enable detailed probing; they help to establish closer rapport with interviewees, thus potentially facilitating access to more sensitive topics. However, they are also open to desirability bias and are only feasible with a sub-sample of large trial populations. They are time-consuming, especially to transcribe and translate, and difficult to analyse in a systematic way because there are no universally accepted criteria for the interpretation of results. They also require highly skilled interviewers, who are often scarce.

#### Self-assessment

It is often argued that self-assessment reduces the risk of desirability bias and therefore generates more accurate reporting of sensitive behaviours. Various methods have been, or are being, tried:

- **Diaries** are usually simple paper documents that participants take home and fill in each time they have sex or use the study product [11]. Because participants keep them at home, diaries can reduce recall bias. However, participants may forget to fill them in, and there is no way of knowing whether they were completed as intended or all at once, just before handing them in. They can also be misplaced or lost and there is no immediate way for participants to seek clarification if something is unclear. Diaries may also be prone to desirability bias because participants know that researchers will be able to link the data to them individually.
- **CASI (computer assisted self-interviewing).** Participants are given a computer or a hand-held electronic device and asked to respond to visual cues on the screen or questions that they hear through headphones [12], [13]. CASI is becoming increasingly popular because it is assumed to reduce desirability bias. This assumption is based on higher reporting of sensitive behaviours in CASI compared to face-to-face interviews [14]. However, while this seems intuitively plausible, there is no clear evidence to support this, and there may be other reasons for the higher reporting.
- **Ballot boxes** (or secret voting). Participants are asked to fill in answers on a form that they then deposit anonymously in a box [15]. This has not been widely used, but it is a simple and cheap method and it seems plausible that participants might be inclined to report more honestly on sensitive topics.
- **Electronic and telephone messaging.** This includes participants using mobile phones, emails or the internet to report behaviour (for example in the Adolescent Trials Network microbicide study ATN-062 (running parallel to the MTN-004 trial) participants will use a computerised phone diary to describe their experiences with the study product [16]). As yet there is no evidence on how such techniques work in practice, but they have the potential of reducing recall bias, though they may also be prone to desirability bias.

Despite their individual merits for measuring adherence and sensitive behaviour, none of these self-assessment techniques are capable of generating in-depth understanding of behaviours or reasons for behaviours.

### Other methods

There are a number of other methods that are used (or could be used) in microbicide and related research to collect behavioural information.

- **Focus group discussions (FGD)** are relatively easy and cheap and have proved popular in collecting qualitative data on acceptability. They can also be used to collect indirect evidence on adherence and sexual behaviour, and they can be a source of respondent-independent data (people reporting other people's behaviour) and of indirect self-report data (respondents talking about themselves in the guise of talking about what “others” do). They are also a rich source of information on community norms and values. The main disadvantage, especially relating to data on adherence and sensitive behaviours, is that they are difficult to verify, may be dominated by a small number of more vocal individuals, and may not give any useful information on the frequency of behaviours or attitudes. They also generate information that tends toward the social norm.
- **Ethnography**. While not an obvious method for collecting information on adherence or sensitive behaviour, and not generally used in microbicide research, ethnography (including informal observation and conversation) can be a rich source of both direct and indirect data.

### Mixed methods

One way of overcoming the disadvantages of individual methods and enhancing the accuracy (and depth) of self-report data is through the combination of different methods. There is a growing methodological literature on mixed method research [17], [18], [19], [20], and even a dedicated *Journal of Mixed Methods Research*. Although there is no precise and universally accepted definition of “mixed methods” the term usually refers to some combination of quantitative and qualitative methods. Mixed methods are used for a variety of reasons:

1. To develop or evaluate study tools and procedures.
2. To examine different aspects of the research question.
3. To broaden the scope of the research.
4. To triangulate results in order to get more accurate data.

Qualitative methods are not commonly used in the context of clinical trials, but when they are this is generally in the form of small ancillary components aimed at collecting data on acceptability. They are also used to inform protocol development and questionnaire design in the early stages of the trial [21], follow up patients' experiences after the trial [22], or for both preparatory and evaluation purposes [23]. Qualitative methods are sometimes used during trials to evaluate trial processes such as informed consent, accrual and retention, usually resulting in suggestions for improvement in future studies [24], [25], [26], or, occasionally, to create a feedback loop between participants and researchers in order to improve processes during the trial [27], [28]. Recently there have been calls to give qualitative methods a more central role in clinical trials [29], [30], [31].

We do not limit the definition of “mixed methods” to the combination of quantitative and qualitative approaches and we consider that the use of different quantitative methods together, or different qualitative methods, could also be described as “mixed method” if they are used in the same project to study the same phenomenon or different aspects of the same phenomenon. We shall also avoid any theoretical discussion about the distinction between “methods” and “techniques”, mixed “methods” vs mixed “models”, and what exactly the term “mixed methods” does or should refer to.

Following this broader definition, there are various examples of the use of mixed methods to collect behavioural data in medical research on sexual and reproductive health. These studies have been concerned with the accuracy of data on sensitive behaviours and have often used biomarkers to validate self-report data. They have had mixed results. For example, one study of men and women attending an STI clinic in the US found that self-reported condom use was not supported by STI incidence [32], while another study, also of STI clinic attendees in the US, found that self-reporting was supported by STI incidence data [33]. In Zambia a study found that couples under-reported unprotected sex when compared to STI incidence and a biomarker (presence of semen) [34], and a study in Tanzania found that self-reports were inconsistent compared to biomarkers, but that in-depth interviews were better than self-completion questionnaires [35].

In microbicide research there is a trend towards experimenting with new methods – particularly CASI – in combination with more conventional ones, such as face-to-face interviews. CASI has been used in three MTN clinical trials in an attempt to get more accurate information on sexual behaviour. In the VOICE trial (MTN-003), a Phase IIb study of Tenofovir vaginal gel and Truvada tablets for the prevention of HIV infection in women, CASI is being used to ask the questions the researchers deem sensitive; at three of the South African Carraguard phase III trial sites CASI was assessed against various STI biomarkers [36]; and in one of the HPTN035 sites in Malawi CASI was compared with face-to-face interviews [37]. CASI is becoming a popular method through which to validate responses from face-to-face interviews, but is also itself being validated against biomarkers. In an example of the former, a CASI survey was conducted within the phase II trial of Carraguard in South Africa to investigate whether participants ever intentionally misled interviewers in face-to-face interviews [38]; and among a subset of women who completed the MIRA diaphragm trial, self-reported sexual behaviour via CASI and face-to-face interviews was validated against a biomarker of recent semen exposure [39].

### Triangulation

Until we have validated biomarkers for all the behaviours that are relevant to microbicide trials, such as adherence, sexual behaviour, condom use – something that does not seem likely in the near future – we will remain dependent on some form of self-reporting for most of these data. And we will also continue to need self-report data to inform us about the details of and reasons for particular behaviours. It is therefore crucial to continue to develop and improve methods so that the data we collect are as accurate as they can possibly be. One way forward is to go beyond the parallel use of different methods to triangulation.

The term triangulation is derived from surveying and navigation, where it refers to finding a position – a fixed point – by getting bearings on different objects. The methodological use of the term is usually traced back to a 1959 article by Campbell and Fiske [40]. Although there is no universally accepted definition of research triangulation, it tends to refer to combining the results of complementary methods in order to get more accurate result [20]. Campbell and Fiske refer to “convergent validation” [40].

This is a simplification, however, and in the literature on social science research methods there has been heated discussion about what triangulation is and is not, and whether it is possible at all [41], [42]. Basically, the controversy revolves around epistemological issues: whether, in the social realm, there is a “fixed point” at all, and whether the fact that method A agrees with method B makes either method more valid. These issues are relevant for a number of reasons. First, while we do not take a relativist position relating to the “truth” of the behaviours we are studying, it is clear that this truth is of a different order to the “fixed point” of the surveyors and navigators, and most of the key behaviours that we try to measure are ambiguous and difficult to define [43]. Second, convergence does not necessarily mean truth: if we collect data on, say, sex acts using different methods and the numbers are the same, this does not necessarily mean that this is what “really” happened.

While a detailed consideration of these issues falls outside the scope of this paper, we are raising them here because we want to make clear that the triangulation model we describe here represents an attempt to move beyond simply comparing methods and trying to work out which is more accurate, toward developing a more composite and holistic picture, while at the same time accepting a necessary degree of uncertainty in the result. Perhaps the term “triangulation” is not the ideal term for this process, given its connotations of precision, but we will continue to use it here for want of a better one. The model described below is based on a model initially developed and used successfully for a number of years to study sexual behaviour change in Uganda [44].

## The Microbicides Development Programme (MDP) Model

### MDP301

MDP is an international partnership set up to evaluate vaginal microbicides to prevent HIV transmission (www.mdp.mrc.ac.uk). A major part of the programme has been MDP301, a multi-centre, randomised, double-blind, placebo controlled trial that aimed to determine the efficacy and safety of PRO-2000 gel in preventing vaginally acquired HIV infection. The MDP301 trial was carried out at three research centres in South Africa and one each in Zambia, Uganda and Tanzania. The enrolment of 9,385 women was completed in August 2008 and follow up was completed in August 2009. Participants were followed up for 12 months post-randomisation, except for Uganda where this was up to 24 months. Participants were recruited from four main populations: women from the general community with access to primary health care facilities (South Africa and Zambia) or who were entitled to primary care either through their employment or their partner's employment (Zambia), women working in bars, hotels, guesthouses and other food or recreational facilities (Tanzania), and women in HIV serodiscordant relationships (Uganda). To participate, women had to be 16 years or over in Tanzania and Uganda, or 18 and over in the other countries, sexually active, HIV negative and not pregnant [45]. The results of the MDP301 trial were announced in late 2009, showing no evidence that PRO-2000 provided protection against HIV infection.

### Feasibility and Pilot studies

Prior to the phase III trial, feasibility studies were conducted at each of the centres to assess retention of participants during 12 months of follow up and to obtain estimates of HIV sero-incidence rates, pregnancy, and condom use in settings where condoms were promoted and provided free of charge and risk-reduction counselling and STI treatment were provided. The feasibility studies also assessed behavioural characteristics of the potential study populations. A pilot study, using placebo gel, followed the feasibility studies, and the results of this were used to inform the final protocol for the phase III trial [45].

A substantial social science component was included from the outset. The main objectives of this were to improve and assess the accuracy of adherence and sexual behaviour data, collect detailed data on sexual behaviour and vaginal hygiene practices, assess participants' comprehension of the study and the informed consent procedures, and assess the acceptability of the product and trial procedures [45].

At the start of the feasibility study it was not clear which methods, apart from the conventional clinical CRFs, would be used and what they would look like. Feasibility and pilot studies facilitated internal discussion and consultation with the study communities, as well as the development and testing of methods and approaches.

Because concerns had been voiced about the feasibility and acceptability of coital diaries in some of the study communities, the social science teams at each of the centres developed different formats and tested these in the study communities during the feasibility study. Key questions were: should the diaries be pictorial or text, how explicit should the pictures be and would this be acceptable, which behaviours should they cover, how detailed should they be. One site (Tanzania) tested five different formats [46]. The result of this process was that the team agreed on a basic generic format of a simple pictorial coital diary, and each site then selected images that were locally comprehensible and acceptable.

One centre (Johannesburg) piloted CASI, in particular relating to sensitive topics such as anal sex, but the results were not very different from those achieved with interviews, and this, together with technical difficulties at the time and the impracticality for large study populations in some of the rural areas, led to the decision not to use CASI. The use of mobile phones was also considered and rejected for similar reasons.

Although the use and centrality of a case record form (CRF) for the collection of behavioural data was assumed from the start, the feasibility and pilot studies enabled it to be developed and tested in parallel to the coital diaries and in-depth interview guides, enabling the comparison of data for individual sex acts.

Another important aspect of the feasibility studies was to investigate the local cultural context and clarify key concepts and terms. This involved identifying key vernacular terms relating to relationships and sexual practices and exploring their meanings. Many of the relevant behavioural terms were highly ambiguous, and this was further complicated by the multilingual nature of some of the study sites. As a result there were often multiple possible translations, none of which reflected the exact meaning of the standard English terms that are used in this type of research. The feasibility and pilot studies facilitated some refining of translations and terminologies.

### Mixed methods and triangulation in MDP301

#### The methods

Different methodological options were assessed and developed during the feasibility study. An effort was made to select methods that were relatively simple and feasible across the different settings, and which had both complementary strengths and different weaknesses (to reduce the possibility that agreement in the results may be a result of sharing the same weakness). These methods were then tested in the pilot study and refined before being adopted in the trial:

- Structured interviews recorded on case record forms (CRF)
- Pictorial coital diaries (CD)
- Semi-structured in-depth interviews (IDI)
- Counting returned gel applicators
- Focus group discussions (FGD)
- Ethnography

This selection combines quantitative and qualitative, self-assessment and face-to-face, and self-report and a more respondent independent technique. The only potential biomarker available at the time was the applicator stain test developed by the Population Council [47], [48], but this had not been validated and it was decided that collecting and counting used and unused gel applicators would provide a more respondent-independent means of verifying self-reported adherence [49].

Focus group discussions with community members and trial participants and ethnography carried out in the study communities and clinics provided additional contextual information. The CRF, CD and IDI were developed in parallel and covered the same topics and the same time period in order to facilitate comparison.

#### The social science component

At each of the six African centres a subset of women was randomly assigned to the social science component of the study, which was responsible for the triangulation. The target sample size for this subset was at least 100 per centre (i.e. a total of 600 women across the trial). This number was thought to be small enough to enable the collection of detailed qualitative data and yet large enough to generate results that could be generalised to the whole trial population. By the end of recruitment we had recruited a total of 725 women (7.7% of the trial population) into the social science subsample.

All trial participants had 4-weekly clinic visits during which they received gel and condom supplies, returned used and remaining unused gel applicators, and were interviewed using a CRF. The visits at weeks 4, 24, 40 and 52 were longer as they included a clinical interview and examination and the CRF interview was more detailed, containing questions about gel use, vaginal washing and other practices, and detailed questions on each sex act during the last week (or four weeks if the participant did not have sex in the last week). The triangulation procedures were linked to three of these long clinic visits, at weeks 4, 24 and 52. (It was felt that it was sufficient to triangulate data early, in the middle and at the end of follow-up and therefore unnecessary to also include these procedures at week 40 as well).

The social science component of the trial was made up of teams at each centre consisting of 4–5 interviewers led by a senior social scientist. The social science component was coordinated centrally to ensure standardised procedures and training.

#### The triangulation process (see Figure 1)

1. **Coital diaries.** Four weeks before the long clinic visits the women randomised to the social science component received a coital diary (CD) in which they recorded their sexual behaviour, gel and condom use, and whether or not they inserted anything other than study gel. During the clinic visit they handed in their CD.
2. **Applicator return.** They also handed in their used and unused gel applicators, and these were counted and recorded.
3. **Clinic CRF interview.** A member of the clinic staff then interviewed them about sexual behaviour and gel use in the last week (or 4 weeks if they had not had sex in the last week) using a structured case record form.
4. **Comparison form.** Shortly after the clinic interview a member of the social science team copied the key information on sexual behaviour, gel and condom use from the CRF and the CD onto a comparison form, which was integrated into the in-depth interview guide. This enabled them to see any inconsistencies at a glance.
5. **In-depth interview.** A few days later a social scientist interviewed the participant, focusing on the same period as the CD and the CRF interview and on the same behavioural and product-related topics, but in a more open and informal manner. Answers to the key questions on sexual behaviour and gel adherence were also noted on the comparison form. During this interview the interviewer also probed to find out the reasons for any discrepancies between the data from different methods, and attempted to establish the most accurate answer in discussion with the participant. The final corrected result was recorded on the comparison form. These interviews were all recorded digitally.
6. **Male partner interview.** Consenting male partners of participants who agreed were also interviewed about sexual behaviour during the same period.
7. **Summary database.** The in-depth interview guide also contained a summary section with pre-coded answers and summary fields so that the interviewer could fill in the major findings during or immediately after the interview. These data, together with key data from the CD and the comparison form, were entered into a summary database that provided quick access to the results in a quantitative format.
8. **Quick feedback.** Where relevant, the information from the above process was fed back to the local clinic teams and to the central Trial Management Group during monthly calls to review progress [1].
9. **Focus group discussions** with trial participants and community members about the gel, the trial, sexual behaviour and related issues were carried out to collect more general information on community attitudes. These were all recorded digitally.
10. **Ethnography.** Observations and informal conversations were carried out in the study communities and clinics. Sometimes these activities were aimed at specific problems that arose during the trial.
11. **Transcription and translation.** The recorded in-depth interviews, FGDs and notes from the informal conversations and ethnography were transcribed, translated, and entered in Nvivo, a software programme for the management and analysis of qualitative data.
12. **Coding and analysis.** Transcriptions were all coded in Nvivo and analysed to bring out more implicit meanings and make comparisons between the study sites, using a Grounded Theory approach [50], [51]. Continuous analysis of the data was carried out on a site level at the different research centres as well as centrally across all sites.
13. **Detailed final qualitative analysis.**

**Figure 1:**
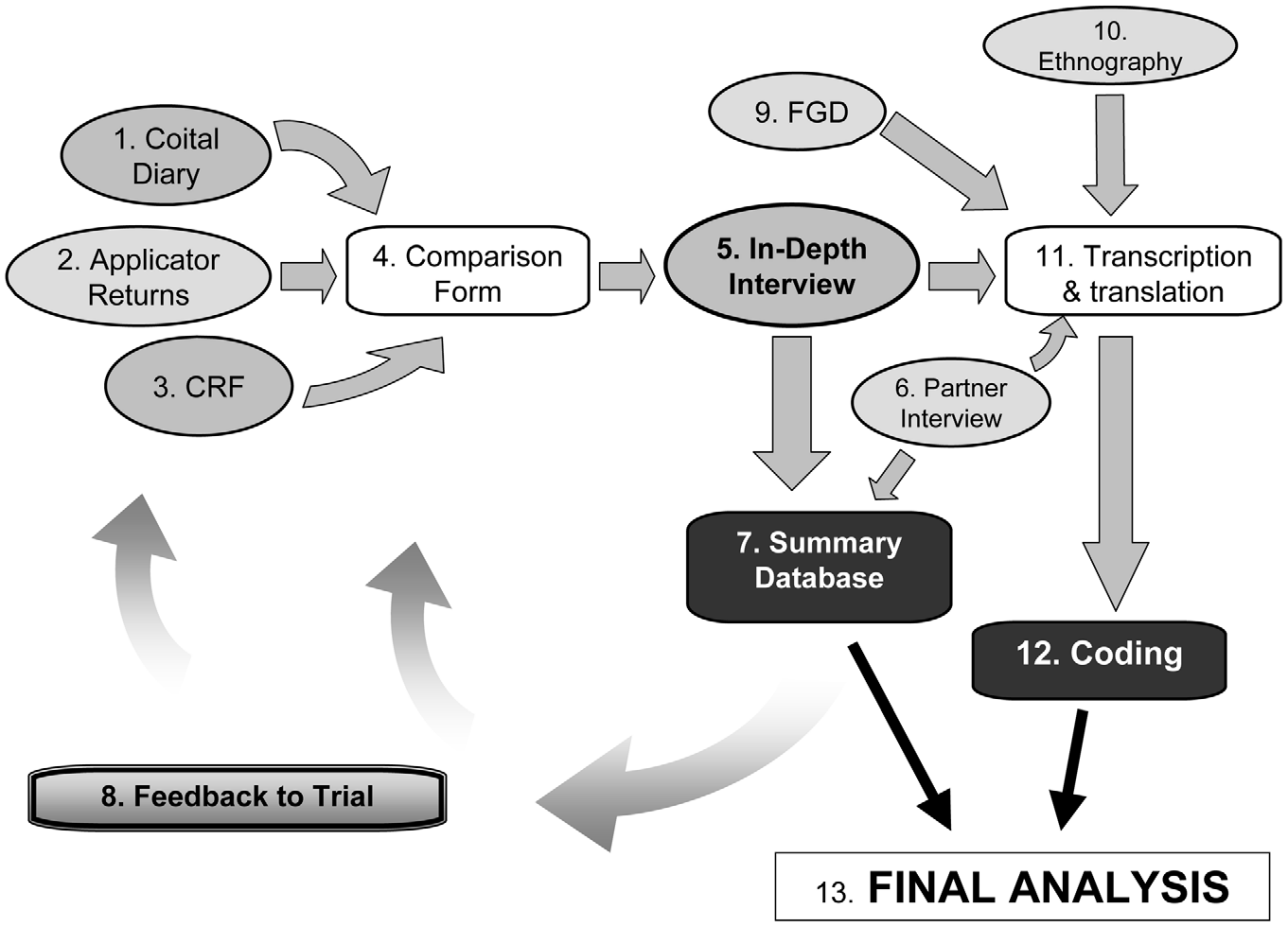
Steps in the triangulation process.

#### The social science data

The final social science dataset consists of 1866 in-depth interviews, most with matching CD, CRF and applicator count data, from 725 women. In addition there are 462 interviews with 244 male partners. There are also 100 FGDs with trial participants who were not randomised to the social science component, 119 FGDs with community members, and extensive ethnographic notes. These have all been transcribed and coded in Nvivo.

## Discussion

In this section we discuss some of the practical issues relating to this approach, looking first at what worked and then considering some of the problems and potential solutions.

### What worked

#### Interdisciplinary collaboration

MDP301 demonstrated that it is possible to integrate a substantial qualitative component into a clinical trial, even one carried out under the stringent criteria required for product licensing. It showed that this approach can greatly enhance the quality and richness of key trial data [1]. This success was partly based on the equality and mutual respect between disciplines, equal representation in management and coordination bodies, equity in the funding for the different disciplines, and the commitment of the funder (DFID/MRC) to this. This does not mean there were no tensions or misunderstandings (see below), but they were easier to solve (or to live with) given the democratic nature of the collaboration.

#### Feasibility and pilot studies

Although feasibility and pilot studies are not essential for a mixed method approach, they did enhance the quality of the collaboration and the data collection tools. In addition to being important from a clinical trial perspective for assessing HIV incidence and retention, the feasibility studies provided space for the development of innovative approaches to collecting sensitive information and the opportunity to integrate these into a coherent methodological whole. They were crucial for understanding the terminologies and meanings that are necessary for developing valid instruments, especially in multicultural and multilinguistic research settings. They were also important for building trust between collaborators from disciplines with very different approaches. Having a pilot study between feasibility studies and trial was important for testing trial and clinic procedures, gel distribution, etc., but also for piloting the combination of methods and the triangulation procedures.

#### The combination of methods

The combination of different but complementary methodological *approaches* – quantitative and qualitative, self-report and respondent independent, and self-assessment and face-to-face – the development of new ways of using and combining existing methods, and the use of triangulation were some of the innovative aspects of this study that worked well.

- **Coital diaries** (although not innovative as such) turned out to be relatively easy and cheap to develop and deploy. Issues of cultural sensitivity and local variations in how images are interpreted were easily overcome by consulting communities in the design. Differences between locations were overcome by using a generic design with local variations in the images.
- **Hybrid in-depth interviews/comparison forms** proved to be a powerful instrument for identifying inconsistencies between data from different sources and for solving most of these through discussion with participants and/or qualitative interpretation of the data. A key aspect of this process was asking participants to explain inconsistencies in their reporting. Initially there had been some reluctance to include this, as it was felt that participants would experience it as “threatening”. However, earlier experience [25] had shown that study participants often welcome the opportunity to correct mistakes and clarify misunderstandings. This was again clearly demonstrated here, showing that it is possible to solve inconsistencies during the study (rather than merely identifying them after the study has been completed) thus increasing the accuracy of the data [1]. The inclusion of tick boxes and summary fields in the IDI guide meant that both qualitative and quantitative data were being collected simultaneously.
- **The summary database** was a quick and efficient means of generating quantitative data from the IDI and for comparing data from different sources, and this enabled rapid feedback to other sections of the trial. For example, information on possible inaccuracies in the CRF interviews were fed back to the clinic and enabled further interview training.
- **Focus group discussions and ethnography** in the clinic and the community complemented the other methods by providing data on the wider socio-cultural context and generated information on problematic issues. For example, FGDs shed light on gel sharing between participants, and when gel use declined at one site informal conversations with participants and analysis of the qualitative data showed that this was due to a bottleneck in supply at the clinic rather than a reduced acceptability. Somewhat unexpectedly, focus group discussions also generated a lot of additional information on topics that we had assumed would not be discussed because of their sensitive nature, such as anal sex.
- **Triangulation**. As mentioned earlier, our aim was to move beyond simply comparing different methods and attempting to work out which is more accurate toward developing a more composite and holistic picture (as well as a more accurate one) based on data from a broad range of methods. There were really two levels to this process: first, “simple” triangulation, in which the results of different methods relating to sex acts, gel use, etc. were discussed with participants and discrepancies analysed and resolved; and second, broader, contextual triangulation, in which the results from individual participants were also related to more general data emerging from focus group discussions and ethnography.

### Some problems and possible solutions

Four main problem areas emerged from, or were highlighted by, this approach.

#### The nature of the topic and the cross-cultural context

Although mitigated to some extent by the ethnographic work during the preparatory phase, the sensitivity of the topics and the lack of fit between the participants' messy descriptions and vague categories and the quantification and ostensibly precise categories of the trialists meant that some ambiguity persisted in the data (for example relating to what should be considered a “sex act”). More ethnographic work could have been done to clarify terminologies in the early stages. Here it is important to focus on how researched communities *use* key concepts, rather than simply asking what the words mean. The conventional approach to questionnaire design, in which local translations of questions are made and then back-translated may simply reproduce standardised, though often inaccurate, matching sets of terms. CRFs for such studies should be designed in a way that more adequately takes intercultural ambiguities into account.

#### Disciplinary assumptions and the clinical trial context

Various tensions arose due to different disciplinary assumptions and epistemologies. We give two examples. First, quantitative medical researchers assume that in order for data to be standardised and comparable, respondents must be asked exactly the same question in exactly the same way. This requires reading the question and related explanatory information verbatim from the questionnaire. Here “the same question” refers to the wording and the delivery. From a qualitative perspective, however, the focus is more on the respondents' interpretation of the questions and what they mean by their answers. In other words, it might be necessary to word and ask questions differently in order to ask the “same” question and get comparable answers. For example, it is clear from the in-depth interviews that, as the trial progressed and participants became familiar with the trial definition of a sex act, the meaning of the questions about sex acts changed for them, while the wording of the CRF questions (and their meaning for the trialists) remained the same. This suggests that getting more reliable data might actually require using more open questions.

Second, although it was accepted by the trialists that a degree of flexibility was necessary in the collection of qualitative data, the relatively inflexible clinical trial culture tended to impinge on this freedom. For example, it was difficult to adjust the in-depth interview question guide during the study because of the assumption that it would then need new IRB approval. It also took many months to get agreement (and then only in some research centres) to carry out additional follow up interviews with participants about suspected gel sharing and dumping, because such interviews were not described in the trial protocol. This lack of flexibility is partly due to the assumptions underlying quantitative research and partly a result of the proliferation of GCP rules and IRB requirements, which are perhaps appropriate for clinical data collection but less so when applied to qualitative behavioural studies.

#### Recruitment and training of interviewers

MDP invested much time and effort in training interviewers, and it is difficult to imagine that more could have been done. However, the triangulation process revealed that many of the inaccuracies that could be traced to the clinic CRF were a result of errors made by the interviewers. This was confirmed when we recorded and transcribed a sample of CRF interviews and compared these with the completed CRFs for the same interviews. A similar problem also bedevilled some of the in-depth interviews, which sometimes left much to be desired with regard to the depth of the probing and the follow-up of potentially interesting topics.

These problems might have been mitigated by more training, and by better quality control (for example through regular recording and comparison of a subsample of interviews, as mentioned above, and the integration of this into a system of ongoing training). But recruitment and selection of interviewers is perhaps more crucial. It should not be simply assumed that with a little interview training nurses and councillors make good interviewers, or that because someone has a degree in social science they are naturally able to do rich in-depth interviews. Good interviewing techniques can be learnt, but some people have more aptitude for this type of social interaction than others, and this should be taken into account when recruiting interviewers (for example by getting applicants to do an interview as part of the selection process).

#### The size of the qualitative dataset and the duration of in-depth interviews

This trial generated the largest set of qualitative data that has ever been collected in a single study, as far as we are aware. Although this had the advantage of enabling us to generate numbers from qualitative data for a relatively large and representative proportion of the trial population (7.7%) across multiple sites, it also brought with it a number of technical problems. For example, existing versions of software packages designed to manage and code qualitative data proved incapable of handling such a large dataset, and the data had to be spread across numerous databases. Qualitative software developers need to work toward increasing the capacity of the databases that are part of their programmes.

Also, in-depth interviews are time consuming to do and, especially, to transcribe, translate and code and, given the large number of interviews, this frequently led to backlogs. However, because only part of the in-depth interview was devoted to the triangulation of adherence and sexual behaviour data, with the rest focusing on other broader contextual issues, it should be relatively easy to separate the triangulation process from the rest of the in-depth interview, which could then be made into a much shorter process involving all trial participants, limiting the rest of the in-depth interview to a smaller sub-sample of participants. This “triangulation interview” could then be the source of the final quantitative trial data on adherence and behaviour. These triangulation interviews could be routinely recorded and a sub-sample transcribed for training and quality control.

### Conclusions

This paper has described the integration of qualitative and anthropological methods and innovative quantitative methods into a large multi-centre clinical trial, and the triangulation of results in order to obtain more accurate data on product use and sexual behaviour. While there are various examples of the use of mixed methods in clinical trials, the Microbicides Development Programme has, as far as we are aware, developed and implemented the most comprehensive combination of mixed methods and triangulation in a clinical trial to date. The study is unique in having integrated these into the trial in order to improve accuracy rather than using them for parallel or retrospective evaluations, and in the way that the qualitative data were collected from a substantial representative sample of the trial population. The key innovative aspect is the identification and resolution of inaccuracies in the data during the study in a process that involved a customised in-depth interview and dialogue between researchers and participants.

It is often argued that this type of research is time consuming, that it costs too much, and that the results are not “objective”. But if it is not done then trialists risk having spent millions of dollars and still ending up not knowing what it was they paid so much to find out. The experience in MDP301 suggests ways of re-thinking how we get a true grip on the most challenging aspect of HIV prevention research: adherence to protocol and to prevention behaviours that require enduring commitment.
